# Case-Only Survival Analysis Reveals Unique Effects of Genotype, Sex, and Coronary Disease Severity on Survivorship

**DOI:** 10.1371/journal.pone.0154856

**Published:** 2016-05-17

**Authors:** Jennifer R. Dungan, Xuejun Qin, Benjamin D. Horne, John F. Carlquist, Abanish Singh, Melissa Hurdle, Elizabeth Grass, Carol Haynes, Simon G. Gregory, Svati H. Shah, Elizabeth R. Hauser, William E. Kraus

**Affiliations:** 1 Duke University School of Nursing, Durham, NC, United States of America; 2 Duke University Department of Medicine, Durham, NC, United States of America; 3 Intermountain Heart Institute, Intermountain Medical Center, Salt Lake City, UT, United States of America; 4 Department of Internal Medicine, University of Utah, Salt Lake City, UT, United States of America; 5 Behavioral Medicine Research Center, Duke University Medical Center, Durham, NC, United States of America; 6 Duke Molecular Physiology Institute, Duke University Medical Center, Durham, NC, United States of America; 7 Department of Biostatistics and Bioinformatics, Duke University Medical Center, Durham, NC, United States of America; Universite de Montreal, CANADA

## Abstract

Survival bias may unduly impact genetic association with complex diseases; gene-specific survival effects may further complicate such investigations. Coronary artery disease (CAD) is a complex phenotype for which little is understood about gene-specific survival effects; yet, such information can offer insight into refining genetic associations, improving replications, and can provide candidate genes for both mortality risk and improved survivorship in CAD. Building on our previous work, the purpose of this current study was to: evaluate *LSAMP* SNP-specific hazards for all-cause mortality post-catheterization in a larger cohort of our CAD cases; and, perform additional replication in an independent dataset. We examined two *LSAMP* SNPs—rs1462845 and rs6788787—using CAD case-only Cox proportional hazards regression for additive genetic effects, censored on time-to-all-cause mortality or last follow-up among Caucasian subjects from the Catheterization Genetics Study (CATHGEN; *n* = 2,224) and the Intermountain Heart Collaborative Study (IMHC; *n* = 3,008). Only after controlling for age, sex, body mass index, histories of smoking, type 2 diabetes, hyperlipidemia and hypertension (*HR* = 1.11, 95%*CI* = 1.01–1.22, *p* = 0.032), rs1462845 conferred significantly increased hazards of all-cause mortality among CAD cases. Even after controlling for multiple covariates, but in only the primary cohort, rs6788787 conferred significantly improved survival (*HR* = 0.80, 95% *CI* = 0.69–0.92, *p* = 0.002). Post-hoc analyses further stratifying by sex and disease severity revealed replicated effects for rs1462845: even after adjusting for aforementioned covariates and coronary interventional procedures, males with severe burden of CAD had significantly amplified hazards of death with the minor variant of rs1462845 in both cohorts (*HR* = 1.29, *95% CI* = 1.08–1.55, *p* = 0.00456; replication *HR* = 1.25, *95% CI* = 1.05–1.49, *p* = 0.013). Kaplan-Meier curves revealed unique cohort-specific genotype effects on survival. Additional analyses demonstrated that the homozygous risk genotype (‘A/A’) fully explained the increased hazard in both cohorts. None of the post-hoc analyses in control subjects were significant for any model. This suggests that genetic effects of rs1462845 on survival are unique to CAD presence. This represents formal, replicated evidence of genetic contribution of rs1462845 to increased risk for all-cause mortality; the contribution is unique to CAD case status and specific to males with severe burden of CAD.

## Introduction

*Even death is unreliable; instead of zero it may be some ghastly hallucination*, *such as the square root of minus one*.*—Samuel Beckett*

The state of gene association science with regard to complex, polygenic phenotypes has been largely characterized by clinically insignificant, poorly replicated findings, even for the strongest candidate genes [1]. A number of factors generate Type I and II errors, biases, and underestimation of association or risk in gene association studies: poor study design and phenotype definition; sampling bias; low power; and unrecognized genetic interactions [2–7]. The study of the genetics of coronary artery disease (CAD) is fraught with these issues [8]. In genetic analysis of diseases with a strong mortality burden, such as CAD, gene-specific survival effects may also impact the associations.

These issues are compounded in genetics research: if a genetic variant predisposes to early death, the genetic risk variant will be ‘culled’ from the population, artificially confounding the relationship between the disease-associated gene and CAD in the surviving individuals. This is a problem akin to attrition bias and length biased sampling. We suspected the following issues to be common: 1) sample demographics underrepresent for high-risk attributes; 2) reduced variance in CAD-related phenotypes result in a reduced risk spectrum; 3) reduced genetic variation; 4) significantly different MAF across age groups, 5) a paradox with survival rates for certain risk alleles. Loss of a genetic ‘risk’ variant due to lethal phenotypes can reduce variance in the data and result in hampered ability to detect genetic associations with disease. Simulations have demonstrated that this is a problem especially in cross-sectional designs [9]; this bias has not been observed using actual data and its impact on genetic associations remains to be seen.

Sudden death is often the initial presentation of coronary disease: 50% of men and 64% of women who die suddenly of coronary heart disease have had *no previous symptoms* [10]. Thus, we have hypothesized a role for survival effects in our case-control gene association studies of coronary artery disease [11]: those with early-onset, very severe disease, and/or greater familial burden are more likely to experience fatal events earlier in life (and before study capture). Because of the differential survival among cases with CAD and unaffected controls, biases resulting from the unobserved high-risk individuals are likely to affect the results of CAD association studies.

We previously described a novel phenotype for survivorship in CAD and provided preliminary evidence of survival-variant genetic effects unique to CAD-diagnosed individuals [11] in a biorepository from the Duke catheterization laboratory [CATHGEN] [12,13]. We demonstrated preliminary evidence of limbic-system associated membrane protein (*LSAMP*) SNP effects on time-to-all-cause mortality unique to sex, CAD case status, and CAD severity in a pilot evaluation of 1,885 subjects [11]. Building on our previous pilot study [11], the purpose of this current study was to evaluate select *LSAMP* SNP-specific hazards for all-cause mortality post-catheterization in a larger cohort of CAD cases and perform additional replication in an independent dataset.

## Results

### Demographics

Demographic and cohort characteristics are presented in Table 1. CAD cases in both CATHGEN and IMHC who experienced events were older, had a greater burden of disease (higher CAD index), lower body mass index (BMI), and a higher frequency of hypertension compared to living CAD cases and to controls. Deceased CAD cases from both datasets also had a lower frequency of hyperlipidemia; this is a paradoxical relation between mortality and hyperlipidemia, previously reported by our group, and likely due to treatment effects or confounders [14] [15]. Deceased participants with CAD from both datasets also had a reduced frequency of smoking compared to living CAD cases; history of smoking was a self-report measure where participants had the option to report “having ever smoked” or “currently smoking” as a positive history and “not currently smoking” as a negative history.

**Table 1:** Subject Demographics in Primary and Replication Datasets.

Characteristics	Primary Dataset (CATHGEN)				Replication Dataset (IMHC)			
	Full Dataset (n = 5566)	Controls (n = 1435)	CAD Cases (n = 2828)		Full Dataset (n = 5872)	Controls (n = 1889)	CAD Cases (n = 3314)	
			Alive (n = 2136)	Dead (n = 692)			Alive (n = 2051)	Dead (n = 1263)
**Age ± SD, (range)**	60.4 ± 12.1 (20‒93)	56.2 ± 12.0 (20‒91)	61.3 ± 10.9 (25‒90)	67.6 ± 11.2 (27‒91)	62.5 ± 13.1 (12‒93)	58.5 ±13.9 (16‒89)	61.6 ± 10.9 (21‒89)	70.7 ± 11.3 (12‒93)
**CAD index ± SD**	32.7 ± 27.5	3.8 ± 8.0	52.2 ± 18.6	58.7 ± 19.8	29.9 ± 26.6	1.52 ± 5.2	47.4 ± 16.5	53.1 ± 18.4
**Male, n (%)**	3468 (62.3)	722 (50.1)	1536 (71.9)	494 (71.4)	3839 (66.2)	977 (51.7)	1610 (78.5)	893 (70.7)
**Caucasian, n (%)**	4086 (73.4)	935 (65.2)	1685 (78.9)	539 (77.9)	5293 (90.1)	1684 (89.1)	1869 (91.1)	1139 (90.2)
**BMI ± SD**	30.0 ± 7.2	30.8 ± 7.8	30.0 ± 6.4	29.1 ± 6.8	29.1 ± 6.1	29.6 ± 6.9	29.3 ± 5.5	28.5 ± 5.9
**Hypertension, n (%)**	3819 (68.6)	861 (60.0)	1533 (71.8)	549 (79.3)	3314 (56.4)	901 (47.7)	1218 (59.4)	877 (69.4)
**Diabetes MellitusType 2, n (%)**	1646 (29.6)	299 (20.8)	678 (31.7)	289 (41.8)	1177 (20.0)	248 (13.1)	429 (20.9)	402 (31.8)
**Dyslipidemia, n (%)**	3442 (61.8)	592 (41.3)	1514 (70.9)	470 (67.9)	3092 (52.7)	653 (34.6)	1375 (67.0)	771 (61.1)
**Smoking, n (%)**	2905 (52.2)	592 (41.3)	1235 (57.8)	410 (59.2)	1153 (19.6)	279 (14.8)	501 (24.4)	271 (21.5)
**Subsequent ICC orPTCA, n (%)**	2031 (36.5)	--	1264 (59.2)	278 (40.2)	1470 (25.0)	--	780 (38.0)	375 (29.7)
**Subsequent CABG,n (%)**	859 (15.4)	--	590 (27.6)	145 (21.0)	1165 (19.8)	--	650 (31.7)	370 (29.3)

CATHGEN = Catheterization Genetics Study; IMHC = Intermountain Heart Collaborative Study; CAD = coronary artery disease; SD = standard deviation; BMI = body mass index; ICC = intracoronary intervention; PTCA = percutaneous coronary angiography; CABG = coronary artery bypass graft.

### Follow-up and Events

In the initial dataset (CATHGEN), the median follow-up time was 1,894 days (5.2 y) and maximum follow-up was 4,302 days (11.7 y). At the time of analysis, 1,164 subjects (20.9%) were deceased at follow-up; 2,828 subjects met criteria for CAD diagnosis with an event rate of 24.5% (*n* = 692) and 1,435 subjects were considered controls, with an event rate of 14.8% (*n* = 212). In the replication dataset (IMHC), the median follow-up was 3,481 days (9.6 y) and the maximum follow-up was 6,362 (17.5 y). At the time of analysis, 1,952 subjects (33.2%) were deceased on follow-up; 3,314 subjects met criteria for CAD with an event rate of 38.1% (*n* = 1,263) and 2,051 subjects were considered controls, with an event rate of 22.4% (*n* = 460).

### Genetic Variation in the Probability of Survival: Initial Analyses & Replication

Our initial analyses (additive models) are reflected in Table 2. Dominant and recessive model results are presented in S1 Table. For the rs1462845 variant, all CATHGEN results are provided for the larger sample of 9,262 participants. For the rs1462845 variant, each addition of the ‘G’ allele in CATHGEN Caucasians with CAD conferred 1.11-times significantly increased hazard of death compared to wild-type homozygous (A/A genotype) carriers, only after controlling for cardiovascular-related covariates (95% *CI* = 1.01–1.22, *p* = 0.032). This result was not replicated in the IMHC dataset (*HR* = 0.98, *95%CI* = 0.89–1.07, *p* = 0.625).

**Table 2:** Hazards of Death by SNP (Additive) in Caucasian CAD Cases.

SNP	Primary CATHGEN Dataset CAD Cases					Replication IMHC Dataset CAD Cases				
	N (MAF)	Gene model^^^		Covariate model^†^		N (MAF)	Gene model^^^		Covariate model^†^	
		HR (95% CI)	*p*	HR (95% CI)	*p*		HR (95% CI)	*p*	HR (95% CI)	*p*
rs1462845	6872 (0.338)	1.08 (0.98‒1.18)	0.125	1.11 (1.01‒1.22)	0.032*	4971 (0.350)	0.97 (0.89‒1.07)	0.567	0.98 (0.89‒1.07)	0.625
rs6788787	4063 (0.152)	0.806 (0.70‒0.93)	0.004**	0.80 (0.69‒0.92)	0.002**	5043 (0.151)	1.03 (0.92‒1.16)	0.634	1.04 (0.92‒1.17)	0.526

SNP, single nucleotide polymorphism; MAF, minor allele frequency; CAD, coronary artery disease; HR, hazard ratio; CI, 95% confidence interval.

^^^Gene model: age, sex, main effect of genotype (additive model).

^†^Covariate model: gene + covariates [age, sex, body mass index (BMI), histories of hypertension (HTN), type 2 diabetes mellitus (T2DM), hyperlipidemia, smoking].

*p < .05

** *p* < .01

A different hazard effect was seen with the rs6788787 variant; the hazards of death in CATHGEN Caucasians were significantly *reduced* for main effects of the gene (*HR* = 0.81, 95% *CI* = 0.70–0.93, *p* = 0.0004) and after controlling for covariates (*HR* = 0.80, 95% *CI* = 0.69–0.92, *p* = 0.002). While these CATHGEN findings were consistent with our pilot analyses of 1,885 CATHGEN subjects [11], these effects were not replicated in the IMHC dataset.

### Sex- and disease burden-stratified analyses & replication

#### Males with severe burden of CAD

**Fig 1:**
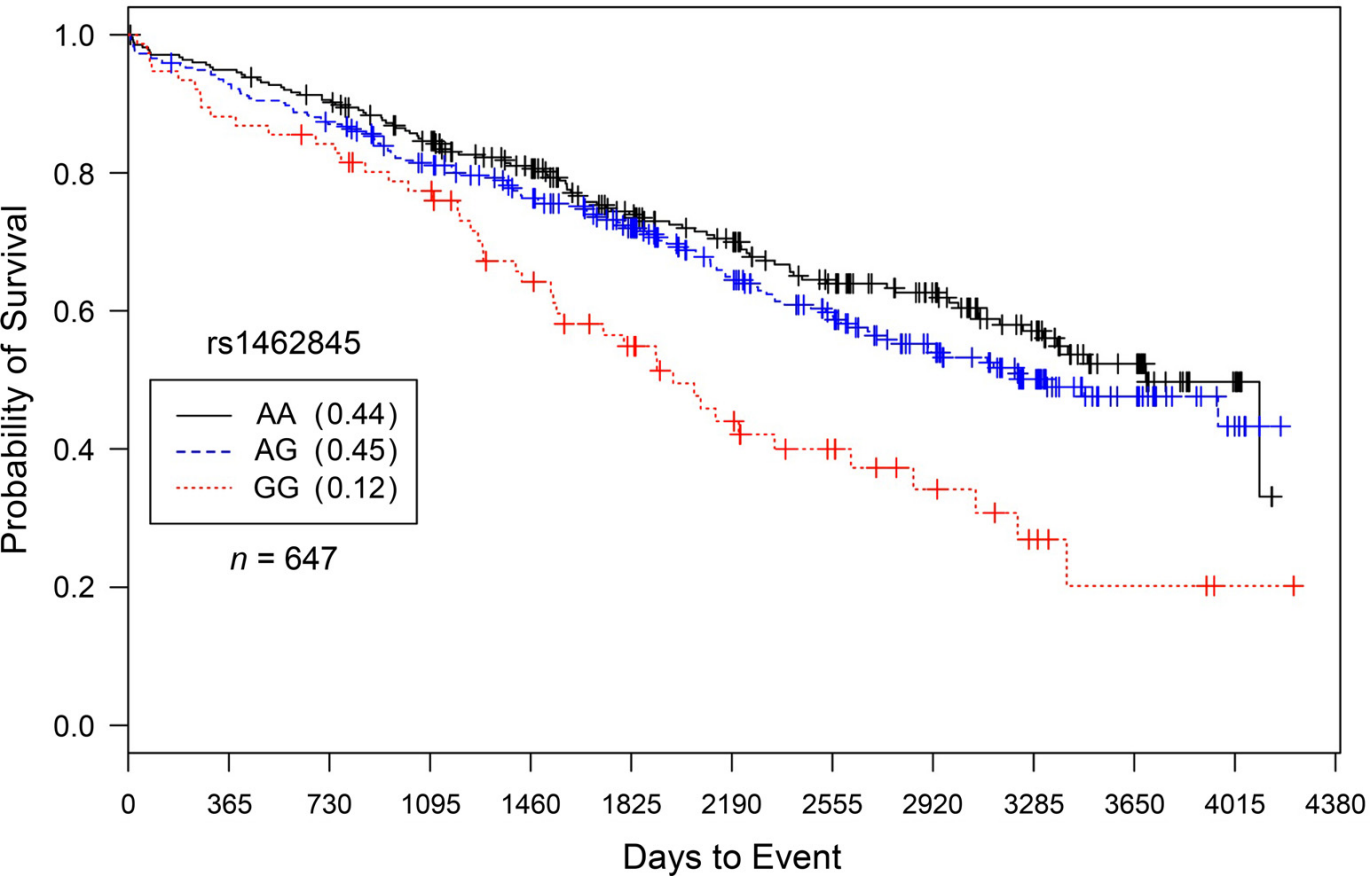
Kaplan–Meier survival curves for CATHGEN males with severe CAD by genotype for *LSAMP* SNP rs1462845. *X*-axis displays the number of days from index catheterization to death (all-cause mortality). *Y*-axis displays the Kaplan-Meier survival probability by genotype. G is the minor allele; AA, wild-type genotype (reference; black curve); AG, heterozygous genotype (blue curve); and GG, risk homozygous genotype (red curve). Hazards of all-cause mortality were significant for each addition of the G risk allele (*HR* = 1.39, *95% CI* = 1.16–1.66, *p* = 0.0003, additive genetic model).

**Fig 2:**
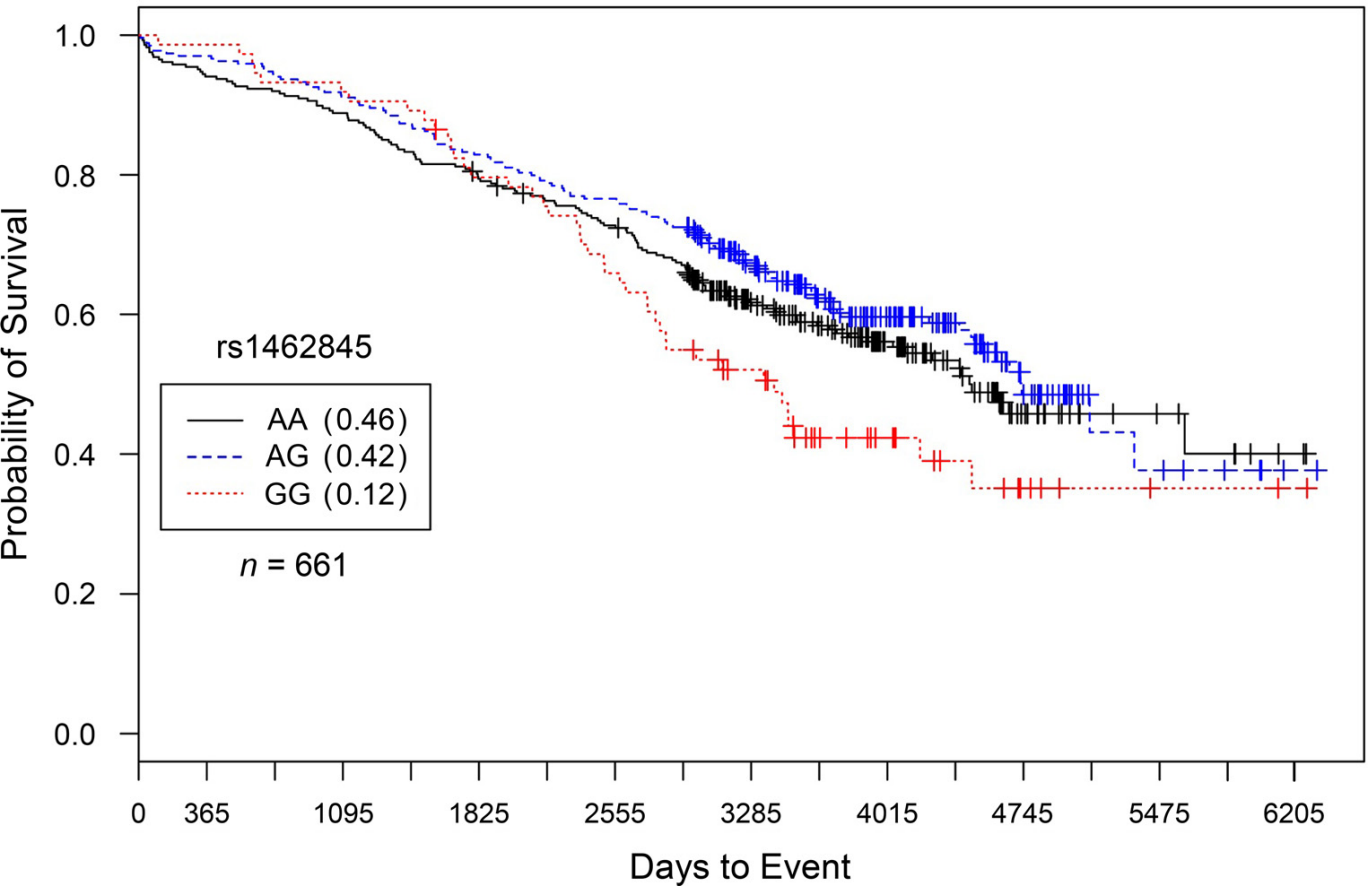
Kaplan–Meier survival curves for IMHC males with severe CAD by genotype for *LSAMP* SNP rs1462845. *X*-axis displays the number of days from index catheterization to death (all-cause mortality). *Y*-axis displays the Kaplan-Meier survival probability by genotype. G is the minor allele; AA, wild-type genotype (reference; black curve); AG, heterozygous genotype (blue curve); and GG, risk homozygous genotype (red curve). Hazards of all-cause mortality were significant for each addition of the G risk allele (*HR* = 1.22, *95% CI* = 1.03–1.45, *p* = 0.0188, additive genetic model).

Results are presented in Table 3 (additive) and S2 Table (dominant, recessive). Caucasian males having at least 3-vessel disease with proximal left anterior descending artery or left-main artery stenosis (CAD Index > 67) had statistically significant increased hazards of death with each addition of the ‘G’ allele for rs1462845 (*HR* = 1.4, 95%*CI* = 1.16–1.66, *p* = 0.000312; Fig 1); this effect persisted even after controlling for cardiovascular-related covariates (*HR* = 1.33, *95% CI* = 1.11–1.58, *p* = 0.00382) and coronary interventions (*HR* = 1.29, *95% CI* = 1.08–1.55, *p* = 0.00456). With the same direction of effect and similar hazard magnitudes, these results for rs1462845 were replicated in Caucasian males with severe burden of CAD in the IMHC dataset (Fig 2) for the main effect of gene and after controlling for covariates (Table 3). In order to estimate the genotype-specific hazards, we conducted a sub-analysis of homozygous risk compared to wild-type individuals (CATHGEN: *HR* = 1.22, *95% CI* = 1.48–3.01 *p* = 3.54 x 10^−5^, IMHC: *HR* = 1.56, *95% CI* = 1.10–2.22 *p* = 0.013) and heterozygous compared to wild-type individuals (CATHGEN: *HR* = 1.18, *95% CI* = 0.907–1.54, *p* = 0.212 IMHC: *HR* = 0.87, *95% CI* = 0.67–1.12 *p* = 0.268). The covariate-adjusted model gave consistent results (S3 Table). Therefore, in both cohorts, the rs1462845 homozygous risk genotype accounts for the increased hazard in the additive model.

**Table 3:** Hazards of Death by SNP (Additive) in Caucasian Males with Severe Burden of CAD.

SNP	Primary CATHGEN Dataset Male CAD Cases, Severe burden (CADi > 67)							Replication IMHC Dataset Male CAD Cases, Severe burden (CADi > 67)						
	N (MAF)	Gene model^^^		Covariate model^†^		Intervention model^‡^		N (MAF)	Gene model^^^		Covariate model^†^		Intervention model^‡^	
		HR (CI)	*p*	HR (CI)	*p*	HR (CI)	*p*		HR (CI)	*p*	HR (CI)	*p*	HR (CI)	*p*
rs1462845	647 (0.35)	1.39 (1.16‒1.66)	0.0003**	1.33 (1.11‒1.58)	0.00181**	1.29 (1.08‒1.55)	0.00456**	630 (0.345)	1.22 (1.03‒1.45)	0.0188*	1.30 (1.09‒1.54)	0.00382**	1.25 (1.05‒1.49)	0.0132*
rs6788787	462 (0.15)	0.77 (0.58‒1.02)	0.0697	0.71 (0.54‒0.93)	0.0148**	0.71 (0.54‒0.94)	0.0177*	637 (0.144)	1.04 (0.83‒1.29)	0.752	0.98 (0.78‒1.22)	0.827	1.04 (0.83‒1.30)	0.7240

SNP, single nucleotide polymorphism; MAF, minor allele frequency; CAD, coronary artery disease; CADi, CAD index; HR, hazard ratio; CI, 95% confidence interval.

^**^**^Gene model: age, main effect of genotype (additive model).

^**†**^Covariate model: gene, age, body mass index (BMI), histories of hypertension (HTN), type 2 diabetes mellitus (T2DM), hyperlipidemia, smoking.

^**‡**^Intervention model: gene, age, body mass index (BMI), histories of hypertension (HTN), type 2 diabetes mellitus (T2DM), hyperlipidemia, and presence of any of the following subsequent interventional procedures: coronary artery bypass graft surgery (CAGB), percutaneous transluminal coronary angiography (PTCA) or stent. (Note: PTCA or stent not included as covariates in IMHC model due to lack of data.)

*p < .05

** *p* < .01

**Fig 3:**
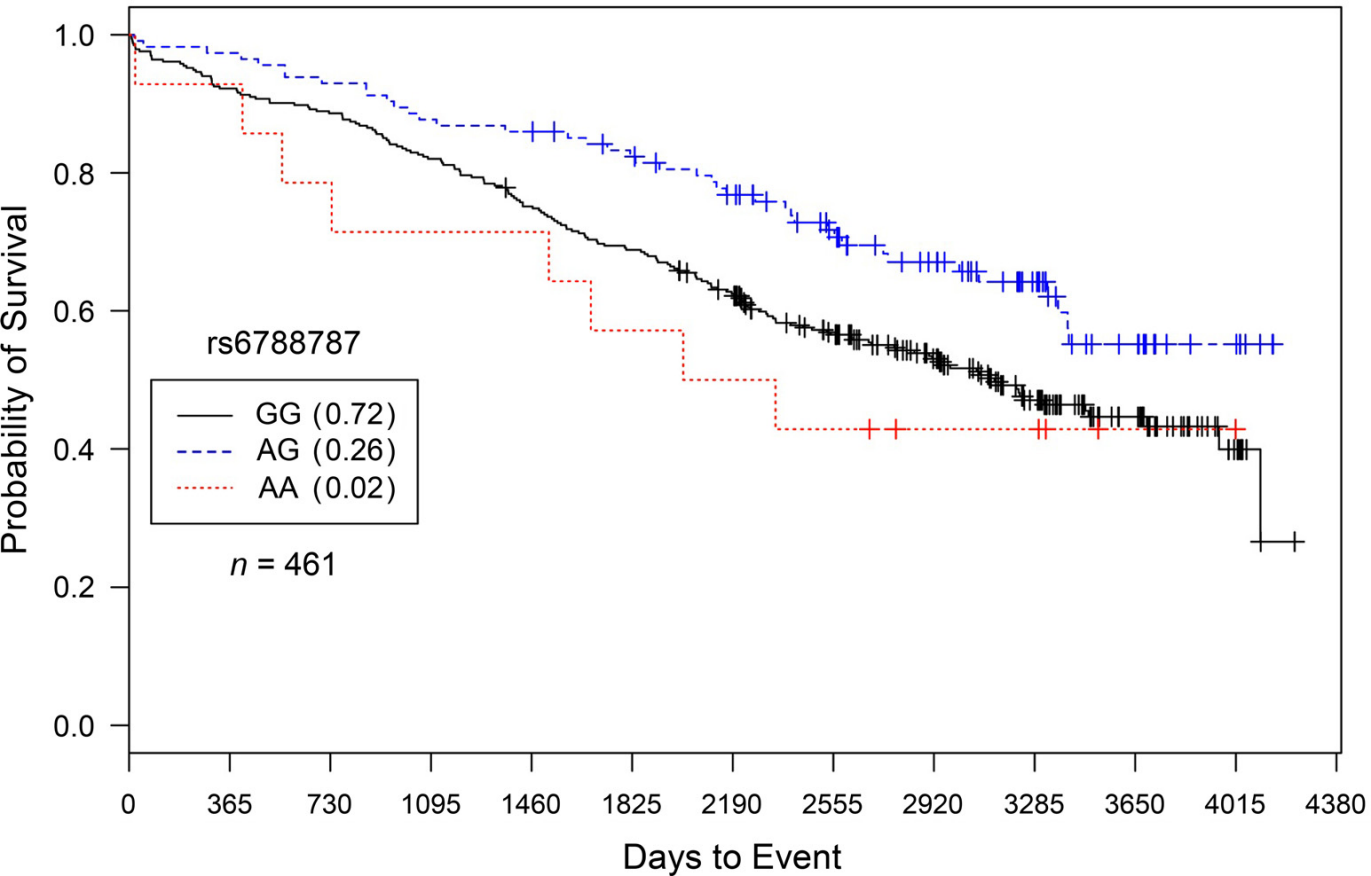
Kaplan–Meier survival curves for CATHGEN males with severe CAD by genotype for *LSAMP* SNP rs6788787. *X*-axis displays the number of days from catheterization to death (all-cause mortality). *Y*-axis displays the Kaplan–Meier survival probability by genotype. A is the minor allele; GG, wild-type genotype (reference; black curve); GA, heterozygous genotype; and AA, risk homozygous genotype (red curve). Only after controlling for covariates was this model significant for reduced hazards of death by genotype (*HR* = 0.71, *95% CI* = 0.54–0.93, *p* = 0.0148, additive genetic model).

**Fig 4:**
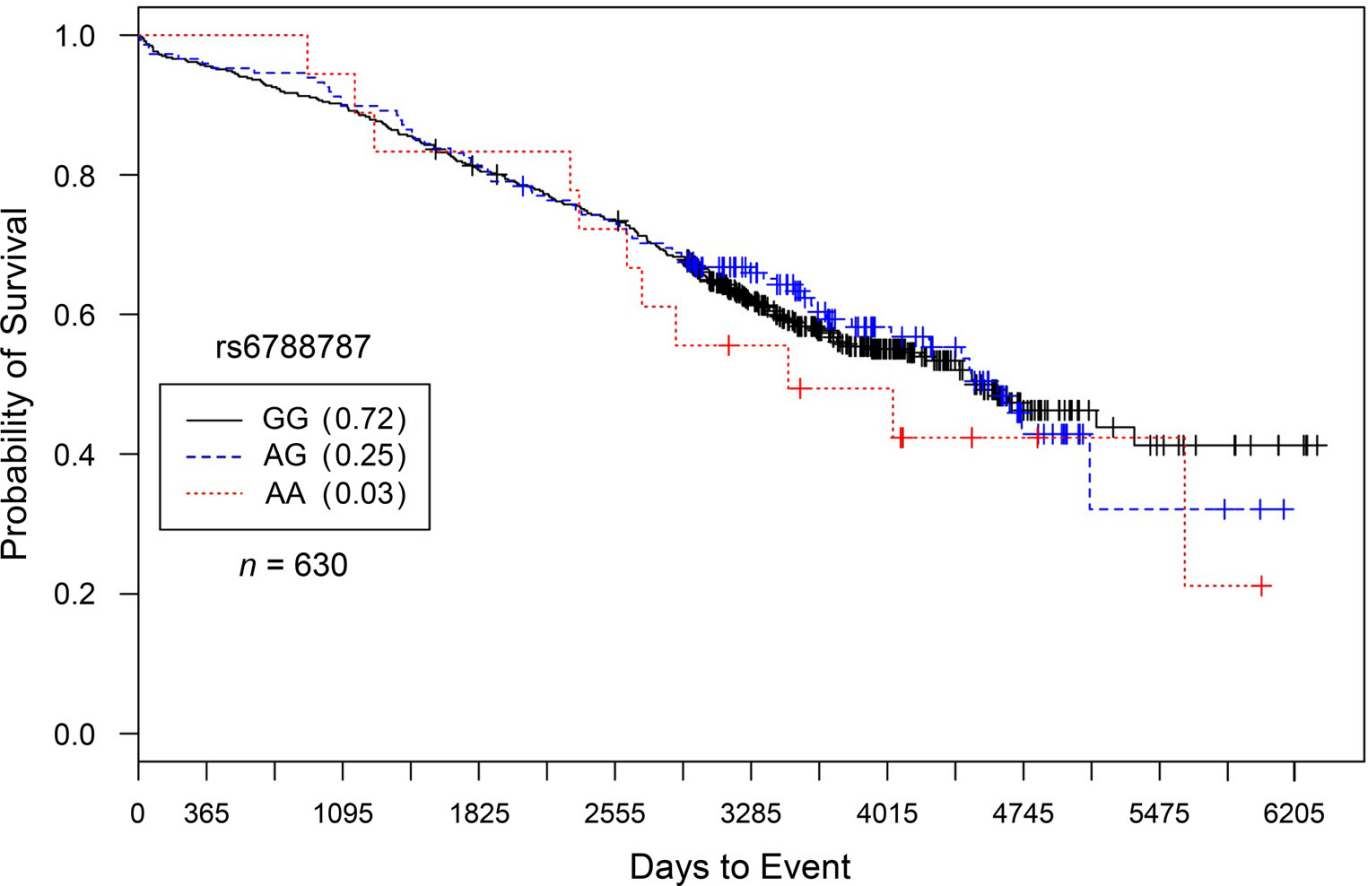
Kaplan–Meier survival curves for IMHC males with severe CAD by genotype for *LSAMP* SNP rs6788787. *X*-axis displays the number of days from catheterization to death (all-cause mortality). *Y*-axis displays the Kaplan–Meier survival probability by genotype. A is the minor allele; GG, wild-type genotype (reference; black curve); GA, heterozygous genotype; and AA, risk homozygous genotype (red curve). This model did not demonstrate significant genotype effects on survival probability (*HR* = 1.04, *95% CI* = 0.83–1.29, *p* = 0.752, additive genetic model).

For rs6788787, in CATHGEN Caucasian males with severe burden of CAD and only after controlling for covariates, each addition of the ‘A’ allele conferred a statistically significant reduction in risk for death compared to G/G genotype carriers (*HR* = 0.71, *95% CI* = 0.54–0.93, *p* = 0.015; Fig 3). These results were virtually unchanged after controlling for subsequent interventional procedures (*HR* = 0.71, *95% CI* = 0.54–0.94, *p* = 0.018; Fig 4). For rs6788787, even after controlling for covariates and interventional procedures, these results were not replicated in the IMHC dataset.

### Analysis of Control Participants

**Fig 5:**
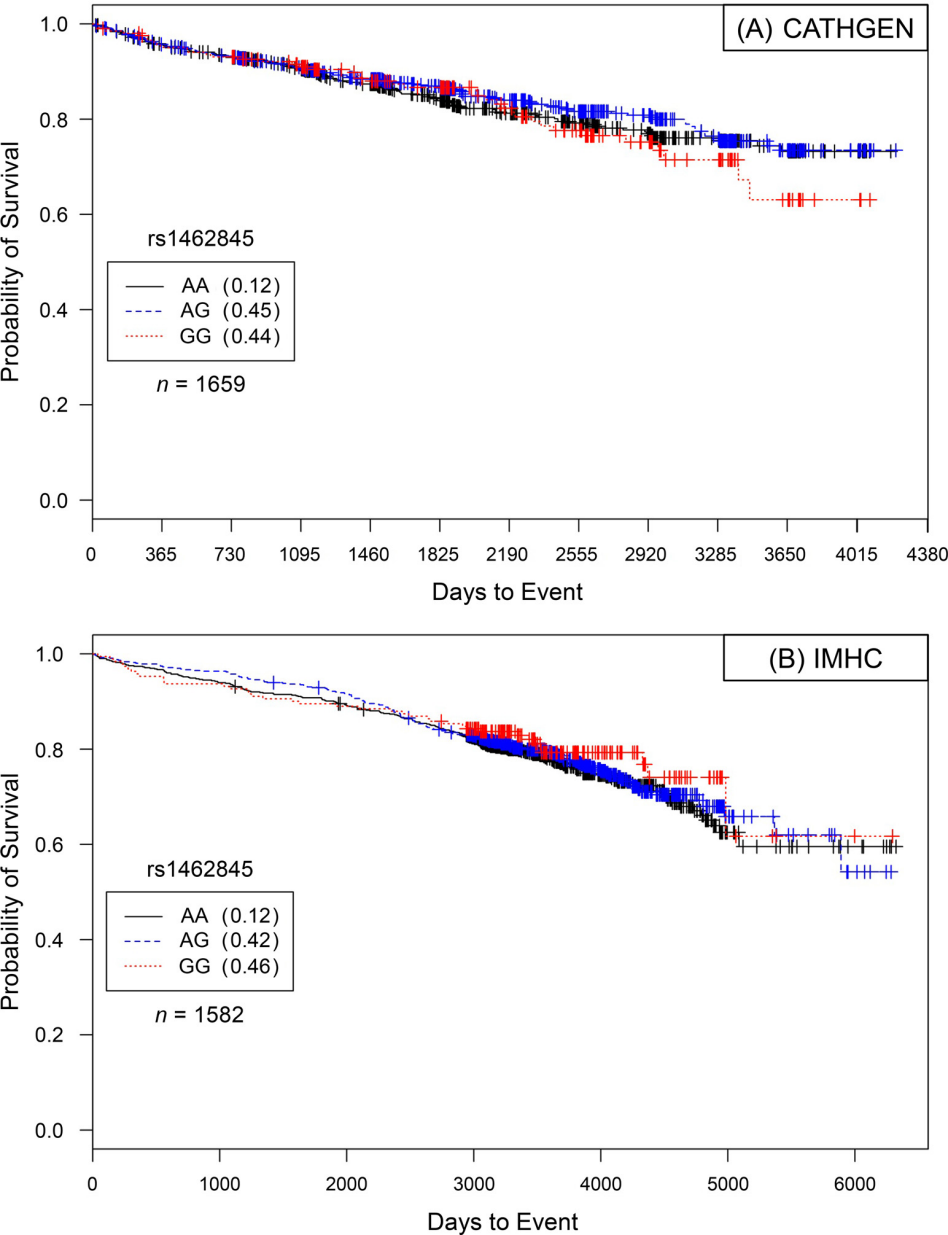
Kaplan–Meier survival curves for 5a) CATHGEN and 5b) IMHC Caucasian control subjects by genotype for LSAMP SNP rs1462845. *X*-axis displays the number of days from index catheterization to death (all-cause mortality). *Y*-axis displays the Kaplan-Meier survival probability by genotype. G is the minor allele; AA, wild-type genotype (reference; black curve); AG, heterozygous genotype (blue curve); and GG, risk homozygous genotype (red curve). No significant differences were found in any statistical modeling (data not shown).

**Fig 6:**
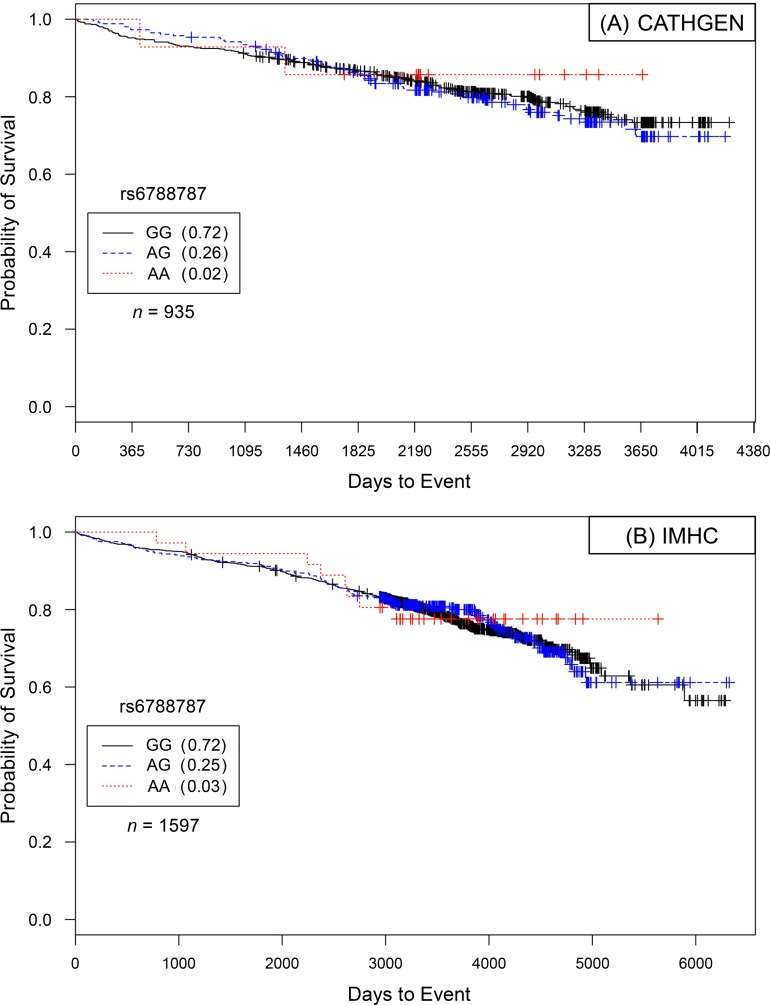
Kaplan–Meier survival curves for IMHC males with severe CAD by genotype for *LSAMP* SNP rs6788787. *X*-axis displays the number of days from catheterization to death (all-cause mortality). *Y*-axis displays the Kaplan–Meier survival probability by genotype. A is the minor allele; GG, wild-type genotype (reference; black curve); GA, heterozygous genotype; and AA, risk homozygous genotype (red curve). No significant differences were found in any statistical modeling (data not shown).

In order to determine if the genetic effects on survival were unique to CAD, participants meeting ‘control’ status (see definition, Study Variables section) were evaluated for survival effects in Caucasians, then in the analysis limited to Caucasian males, controlling for the stated covariates. None of the models conducted in the control groups showed significant genotype-specific hazards (Table 4, Figs 5 and 6).

**Table 4:** Hazards of Death in Caucasian Control Subjects.

SNP	Primary CATHGEN Dataset Controls				Replication IMHC Dataset Controls			
	All Controls		Male Controls		All Controls		Male Controls	
	N	Gene model^^^, *p*	N(MAF)	Sex stratified model^†^, *p*	N	Gene model^^^, *p*	N(MAF)	Sex stratified model^†^, *p*
rs1462845	1435	0.92	722 (34.5)	0.49	1889	0.83	977 (34.9)	0.49
rs6788787	1435	0.67	722 (15.8)	0.93	1889	0.77	977 (14.4)	0.93

SNP, single nucleotide polymorphism; MAF, minor allele frequency; HR, hazard ratio; CI, 95% confidence interval.

^**^**^Gene model = age, gene as covariates;

^**†**^Sex stratified model = age, gene as covariates for male control subsets.

*p < .05

** *p* < .01

### Power

Post-hoc power analyses were conducted using the Cox score test described by Owzar and colleagues; without the limitations of the contiguous alternative assumption, this rigorous approach for determining power for gene-based survival analyses incorporates information about our censoring distribution; risk allele model (additive); and risk allele distribution among both cohorts [16]. Results are reported in Table 5.

**Table 5:** Post-hoc Power Analyses.

SNP	Dataset	MAF	Effect Size (HR)	Event Rate	N	β
rs1462845	Primary	0.35	1.39	0.245	​647	0.808735
rs1462845	Replication	0.35	1.22	0.381	630	0.560207
rs6788787	Primary	0.15	0.77	0.245	462	0.236423
rs6788787	Replication	0.14	1.04	0.381	637	0.064013

SNP = single nucleotide polymorphism, MAF = minor allele frequency, HR = hazard ratio, N = sample size, β = power estimate.

## Discussion

We present replicated evidence of SNP-specific effects on survival probability unique to Caucasian, male, CAD patients with severe burden of disease. Our analysis was adequately powered (Table 5) to detect such effects in CATHGEN (*b* = 0.81) and moderately powered in IMHC (*b* = 0.56). Our *a priori* SNPs are downstream from the limbic system-associated membrane protein gene (*LSAMP*), a gene that has previous associations with late-onset and severe left-main CAD [14]. They are both intergenic SNPs with no previously documented evidence of protein changes or association with cardiovascular mortality. These SNPs were not the same SNPs identified as risk SNPs for age-dependent atherosclerosis (e.g., early- or late-onset CAD) in traditional case-control analyses of genetic associations with CAD [13,17–21]; nor did the age-dependent atherosclerosis risk SNPs demonstrate differential survival (data not shown). This suggests either: the effect of variation in *LSAMP* on CAD and mortality is complex, or there is an ungenotyped genetic variant.

*LSAMP* is known to mediate neuronal cell-cell adhesion [22] and has been implicated as a tumor suppressor gene due to its: decreased expression of tumors compared to normal samples; overexpression inhibiting tumor cell proliferation [23] [24]; expression as a negative predictor of outcome in patients with epithelial ovarian cancer [24]. In human aorta and smooth muscle cell experiments less expression of *LSAMP* in arterial wall SMCs confers a higher risk for developing atherosclerosis through promoting SMC proliferation [19]. Taken together, these results support a potential link between *LSAMP*, survival, and CAD, perhaps through pleiotropic effects related to a yet-identified comorbidity (such as cancer). In studies of age-of onset for CAD and cancer risk, such an effect has been demonstrated in the Framingham Heart Study for the APOe-2/3 polymorphism [25]. Further investigation would be required to test this hypothesis for *LSAMP*.

Other studies have demonstrated SNP-specific risk for cardiovascular-related death. In 2013, investigators of the GRACE Genetics Study [26] evaluated 23 susceptibility loci for CAD and their association with recurrent MI or cardiac death; a variant upstream of *ABO* (rs579459) was significantly associated in initial and replication analyses with both endpoints in 2,099 acute coronary syndrome patients followed for a median five years. In studies of CAD and chronic obstructive pulmonary disease, *ADAM33* was related to both all-cause and cardiovascular-related mortality in 1,390 subjects followed for 18 years [27]. In the MASS-II clinical trial of CAD and preserved left ventricular function, the 9p21 variant rs2383206 was associated with higher incidence of all-cause and cardiac-related mortality in 611 patients with established multi-vessel CAD [28]. The minor alleles of angiotensinogen (*AGT)* SNPs rs1926723, rs11122576, and rs6687360, and angiotensin-1 converting enzyme (*ACE)* SNP rs4267385 were associated with increased mortality in 1,186 coronary patients followed for a median three years [29].

A multitude of genetic variants have been associated with increased odds of greater atherosclerotic disease burden and CAD lesion progression. Most notably, having any copy of the T allele for rs7903146 (a common variant in *TCF7L2* candidate gene for type 2 diabetes) conferred increased risk of multiple-vessel obstructive lesions, greater burden of atherosclerotic disease, and increased incidence of death among 889 subjects referred for cardiac catheterization and 559 MASS-II trial participants followed prospectively over 5 years [30]. While the authors’ results were most significant in non-diabetics, we note this study because of the sampling approach for at-risk individuals referred for cardiac catheterization that is similar to the CATHGEN and IMHC studies we evaluated.

Genotype- and sex-specific variation in mortality has also been described by others. *CYP19A1* polymorphism -81371 C>T significantly interacted with sex to predict primary endpoints death, nonfatal MI, and nonfatal stroke among 568 acute coronary syndrome patients and 619 subjects from a randomized, controlled trial of hypertension followed for 3 years [31]. Specifically, male T-allele carriers had significantly greater mortality likelihood; female carriers exhibited non-significant reduced hazards of death [31]. A more recent, prospective investigation of 114 Turkish patients (54 males) with stable angina or angina equivalent symptoms revealed that “LL” carriers of the *PON1* L55M polymorphism had a higher prevalence of atherosclerotic disease burden (Gensini score ≥ 20), but only female LL carriers had statistically significant association with disease severity [32].

We observed an unexplained, significantly reduced hazard of death for rs6788787 in an additive genetic model in CATHGEN; even after controlling for cardiovascular and interventional covariates this was not replicated in the IMHC dataset. There may be several limitations to the study that account for the lack of replication. As the minor allele frequency was the same across cohorts (0.15 in CATHGEN and 0.145 in IMHC), cohort-specific effects are unlikely due to genetic variation. We only included participants in the model that did not have missing variable data, so we evaluated missing data to determine whether this offered an explanation for lack of replication. Most importantly, neither cohort of males with severe CAD had missing event or follow-up data. Missing data on BMI were negligible between both studies (less than 1% missing data in CATHGEN and approximately 2% in IMHC). No other variables in the gene and covariate models had missing data, leading us to believe that missing data differences do not contribute to our inability to replicate the rs6788787 variant. We did observe a significant amount of missing data in CATHGEN for the ‘intervention’ variables (percentage of missing data for subsequent ICC was 59.7%, PTCA, 62.6%, and CABG, 67.3%). In IMHC, CABG and percutaneous intervention data had less than 1% missing fields; however, IMHC had 100% missing stent data (field not collected). Such cohort differences in missing interventional data could explain the lack of replication for the model that controlled for interventional procedures. We have previously observed replicated interactions between smoking behaviour and genetic effects of the *CDGAP* and *KALRN* genes on CAD [33] in our two cohorts. Therefore, we conducted smoking-stratified and gene-by-smoking interaction analyses for rs6788787 (data not shown); there was no significant effect modification by smoking. Other unidentified confounders may be influencing our inability to replicate the reduced hazards for this variant. Furthermore, we are unable to control for medication effects that may unduly influence the survival estimates.

Post-hoc power estimates for the rs6788787 variant demonstrate reduced power across both cohorts, influenced by lower MAF and less events in smaller sub-strata groups (*n* = 647 and 661 in CATHGEN and IMHC, respectively (Table 5). As we have previously hypothesized [11], the high rate of sudden cardiac death as the initial presentation of CAD may contribute significantly to a reduction in CAD-related risk alleles in the population; this may influence the ability to adequately detect genetic associations for CAD. We currently do not have data to support an increased minor allele frequency for these *LSAMP* variants in a sudden cardiac death population; however, others have demonstrated this *in silico* [9]. Simulations show up to a 20% reduction in odds ratios for genetic association with highly lethal disorders (including CAD); interestingly there are greater erosion of effects for populations limited to ages 75 or older. The authors formally describe an anticipated age-dependent drop in minor allele frequency in the population as one helpful indicator of gene-associated survival bias [9]. We did not observe differences in MAF estimates in dbGAP with a range of CEU MAF of 0.13–0.15 for rs6788787 and 0.29–0.31 for rs1462845. Similarly, using a moving average approach described by Payami and colleagues [34], we did not observe a qualitative decrease in MAF across age deciles (S1 and S2 Figs). In our previous work [11] we hypothesized that evaluation of probability estimates of survival, stratified by genotype categories (via Kaplan-Meier estimation) for CAD candidate genes may provide a method to identify potential survival bias [11]. We propose that an artefactual gene-dose association with survival risk is a potential indicator of survival bias. We observed consistent evidence for an effect of the rs6788787 homozygous risk genotypes relative to wild type genotypes. We observed the potential for better survival in individuals with the heterozygous risk genotypes compared to wild type in both cohorts; however, this relation was not statistically significant. The presence (and inconsistencies) of this phenomenon in both cohorts may be suggestive of survival bias; however, a larger sample size would be required to provide proof of this effect. A prospective study starting observations at a young age, and with meticulous CAD phenotyping and mortality follow up, would be required to truly evaluate a survivorship in CAD relationship [11].

In carefully considering our results and limitations, it is not wholly clear why only certain SNPs in the same gene exert survival effects in CAD and not others, especially as 17 *LSAMP* SNPs were initially tested in the pilot [11]. We did evaluate SNPs among other genes identified in the same linkage peak for age-of-CAD onset phenotypes on Chromosome 3 in our pilot but results of those variants were insufficient to warrant further attempt at replication. More extensive evaluation of *LSAMP* and markers in LD with *LSAMP* may prove useful to understanding the relationship between this gene, survival and CAD.

Refining genetic association results and improving replication rates continue to be priorities in the field of complex disease genetics. In complex phenotypes that are both lethal and age-dependent, survival effects may unduly influence association and replication [9]. Genotype-specific survival effects should be characterized in order to determine whether they pose an erosion of effect size or confounding in genetic association models. This study provided an approach for the design, characterization, and evaluation of survival effects in CAD cases and controls. As others have only simulated survival effects in this context [9], we demonstrate significant survival impact on *LSAMP* genetic association in a clinically-significant CAD phenotype using real-world data. This information would be helpful in any future genetic association analyses regarding these specific *LSAMP* variants, in terms of adjusting for known survival-mediated genetic effects. Rather than using simulated estimates of survival-variant effects to approximate potential erosion of effect sizes [9], our data suggests that survival effects within each cohort and for each gene variant (even within the same gene) may be unique and more accurately defined with our approach. Improving the detection and accuracy of survival effects among genetic variants could be a pivotal step forward in improving the signal-to-noise ratio in genetic association of complex disease. Once the uniquely-defined survival effects among genetic variants are characterized (such as, variation in magnitude and direction of hazard ratios, and variation in survival effects among either cases or controls), their unique effects can be minimized by study design [11] or statistical methods [9]. Novel and refined approaches and methods to address such unique survival effects in genetic association is an important topic for future study.

### Lack of Significance in Controls

To specifically characterize evidence of SNP-specific effects on survival in people already diagnosed with CAD, we stratified our analyses by CAD case status [11]. In comparison to significant survival curves by genotype among CAD Cases (Figs 1 and 2 for rs1462845; Fig 3 for rs6788787), the control participants have a lack of distinction between survival curves by genotype (Figs 5 and 6). We wish to note that the Kaplan-Meier graphs are presented with the same y-axes scales for cases and controls in order to most accurately compare patterns among the survival data. The lack of significance in controls across all genetic and statistical models (Table 4, Figs 5 and 6) suggests that the genetic (SNP) effects on survival are unique to the context of clinically appreciable CAD. Perhaps genetic mechanisms related to these SNPs are only activated for mortality risk when significant coronary morbidity is evident. The functional contribution of these SNPs to survival requires further study.

### Limitations

The studied SNPs may not be the causative variants for the associations with increased hazards of death (rs1462845) or improved survival (rs6788787); they may be proxies for other markers in LD. Most likely due to lower event rate compared to the adequately-powered CATHGEN dataset, the replication dataset had slightly reduced power for rs1462845 analyses. We had limited power to detect significant hazards in rs6788787 in both cohorts, most likely due to the combination of low minor allele frequency (~15%) and low event rates. Related to our interest in characterizing CAD-specific survivorship, we acknowledge here, as previously discussed in depth [11], that use of study enrollment (coronary catheterization) as “time 1” does not likely reflect the most accurate start-time for initiation of the coronary disease process. Nonetheless, it is a common time point for CAD incidence, was the earliest time point available for these biorepositories; and, makes our characterization of survival effects generalizable to real-world, common genetic association studies. Similarly, the use of all-cause mortality as our time-to-event endpoint to characterize survivorship in CAD presents a limitation. Both datasets used in our analyses (CATHGEN and IMHC) are biorepositories for which follow-up of events are adjudicated through vital events databases that do not require the specific cause of death. CAD-diagnosed participants in this study may have died of non-cardiac causes, limiting our inferences around CAD-specific survivorship. The ideal endpoint would be CAD-related mortality; this would require exact cause of death—best ascertained at autopsy.

With regard to the Cox models, differences in the follow-up schedules and event adjudication methods between both the initial and replication studies present limitations. The between-study differences in total follow-up time and follow-up intervals may limit replication of some survival effects and lead to hazard ratio differences. However, the participant ascertainment methods and measurement of variables (demographic, clinical, and non-mortality events) are nearly identical between studies; this makes the IMHC dataset a sound and reasonable replication source. Survival bias due to left-censoring could also affect the outcomes of these time-to-event analyses. Specific to this study, we are unable to capture genetic data on those who experience sudden cardiac death prior to potential recruitment (sudden death is the initial presenting symptom in 20–60% of people with CAD [35] [10]) or who refuse coronary catheterization. These are two factors that we could not reasonably overcome. In addition, right-censoring in both datasets presents a common limitation in the Cox model, as not every participant has experienced death during the follow-up period; 75.5% of CATHGEN cases and 61.9% of IMHC cases were alive at last follow-up. The semiparametric Cox regression accounts for right-censoring [36]. Right- censoring due to loss to follow-up was not an issue, as we excluded participants who were lost to follow-up for this project.

While we were able to control for multiple covariates that are known predictors of mortality in CAD (age, histories of hypertension, diabetes, hyperlipidemia, and smoking, and coronary interventional procedures), we are unable to control for the treatment effect of medications. Aspirin, statin and beta-blocker medications are independent predictors of survival in cardiovascular disease and as such they are recommended first-line drugs for CAD-diagnosed patients [37]. In our subset of CAD-diagnosed CATHGEN participants, not all subjects had sufficient medication data to allow for inclusion as covariates. Medication treatment effects could be explaining the improved survival probabilities observed with the rs6788787 SNP. However, given that a majority of cases are likely to be taking at least one first-line cardiac medication, it could be argued that the significantly increased hazards of death with variation in the rs1462845 SNP is observed in spite of medication treatment. Additional studies are needed to determine whether and how pharmacologic treatment effects may impact genetic variation in survivorship with CAD.

## Conclusions

We set out to characterize survival-variant genetic effects for *a priori* CAD candidate genes in two existing studies of symptomatic, CAD-diagnosed individuals from two different regions of the U.S. Survival probability differed by *LSAMP* SNP rs1462845; there was replicated SNP-specific risk for all-cause mortality in symptomatic, CAD-diagnosed males with severe burden of CAD. Alternatively, improved survival in the context of CAD for rs6788787 was not replicated with IMHC data. We observed disease-severity-, sex-, and cohort-specific survival probability patterns. Such effects are consistent in the literature; addressing them comprehensively may improve gene association replications.

Using Kaplan-Meier analysis, we observed evidence for potential survival bias present in the association between rs6788787 and CAD; these were initially present in the larger datasets, but became more prominent in the stratified analyses of males with severe burden of disease. In contrast, the rs1462845 variant demonstrated significant risk effects on survival with the expected, traditional gene-dosing patterns; this variant may represent a true candidate for marking CAD survivorship. These CAD candidate genes represent significant advancement in the identification of genetic effects on survival in the context of coronary disease.

While the clinical significance of these findings is limited, this approach has immediate applicability for identifying survival-specific CAD candidate genes for future investigation and for refining genetic associations. Evaluating genetic variants for survival effects and potential survival bias in the context of clinically appreciable CAD can be valuable for uncovering potential explanations for lack of association or replication among various datasets. Future genetic associations of CAD should consider testing their gene variants for significant survival effects and where applicable, adjust their studies and methods to control for survival effects.

### Clinical Relevance

Every minute, someone dies of coronary disease in America [10]. The ratio of deaths per year to incident cases of stable angina is roughly equal (~400,000:500,000). Clinically, we are poor at predicting survival in coronary artery disease [38]; accurate estimation of survival outcomes is difficult, at best. Evidence has accumulated for the identification of genetic determinants of mortality in complex (non-Mendelian) diseases such as sudden cardiac death [39,40]; acute coronary syndrome [26,41]; cardiac-related death and all-cause mortality [27,28]; as well as all-cause mortality after acute coronary syndromes [42] [43]. These studies present candidate markers for events in samples with those at-risk but largely undiagnosed for CAD. The genetic contribution for mortality events in people with clinically diagnosed CAD has focused primarily on genetic variation in post-intervention/post-treatment outcomes (such as mortality risk in post-CABG). There is great potential benefit for an increased understanding of whether there are unique genetic markers of survivorship in clinically diagnosed CAD.

We have observed that variation in one CAD candidate gene—*LSAMP*—displays differential effects on survival two CAD case-control studies. Further, different SNPs within this same gene exert distinct effects on survivorship in established CAD populations; there may be allelic heterogeneity among two *LSAMP* SNPs: one variant conferred increased (rs1462845) while the other decreased risk for death (rs6788787) in the presence of CAD-diagnosed males with severe burden of coronary disease. If not confounded by survival bias, survival-variant genes may lead to improved understanding of the genetic contributions to survivorship in CAD. Identification of risk-factor mediated genetic effects—such as demonstrated here with sex- and disease-severity—may help to explain differential outcomes in patient subsets and may lead to more informed clinical decision-making for CAD patients in the future.

## Materials and Methods

### Study Design and Sample

We performed retrospective analysis of longitudinal event data from two separate cardiovascular genetics biorepositories from the Southeastern U.S. (Catheterization Genetics Study; CATHGEN) [13]; and, from the Western region of the U.S. (Intermountain Heart Collaborative Study;IMHC) [44]. Both biorepositories recruited pateints presenting to the cardiac catheterization lab (regardless of disease status) for a cardiovascular genetics study and biorepository in which medical history, clinical data, and biological samples were collected. We genotyped the candidate *LSAMP* SNPs in 5,566 consecutive CATHGEN samples and 5,872 IMHC samples for replication. For the rs1462845 variant in *LSAMP*, genotyping was available for an additional 3,696 subjects for a total of 9,262 consecutive CATHGEN individuals.

All referenced studies (primary CATHGEN and Utah IMHC studies and secondary analyses) were approved by the Duke University Medical Center (Durham, NC) and the Intermountain Healthcare (Salt Lake City, UT) Institutional Review Boards. All participants provided informed consent for participation that included biobanking of genomic data, collection of clinical data, and future use of data as approved by primary study investigators.

### Study Variables

All study variables were retrospectively collected from the CATHGEN database. Based on the results of clinical catheterization at time of study enrollment, CAD cases and non-CAD controls were identified. CAD cases were defined as having at least one major epicardial vessel having at least 75% stenosis on coronary angiography at enrollment (Duke CAD index >23) [13]. For the CATHGEN study, controls were defined as having no clinically appreciable CAD (Duke CAD index < 23 and number of significantly obstructed vessels = 0), corresponding to no major epicardial vessel with more than 74% occlusion as demonstrated by coronary angiography at enrollment, and no documented history of cerebrovascular or peripheral vascular disease, myocardial infarction, organ transplant, or interventional or surgical coronary revascularization (coronary artery bypass graft) procedures at enrollment [13]. IMHC controls were defined by the same criteria, except data were lacking for intracoronary interventions (stent or angiography). Cardiovascular risk factors defined at enrollment and included as covariates were: age; sex; body mass index (BMI); histories of smoking, type 2 diabetes, hyperlipidemia and hypertension; presence of subsequent coronary artery bypass graft surgery (CABG); and, presence of subsequent percutaneous transluminal coronary angiography or stent placement (PTCA or stent; included as a covariate in CATHGEN analyses only). Subjects were excluded from analyses if they had missing data for any of the variables in the models.

### Follow-up and Events

The primary endpoint was time (days) from enrollment (corresponding to time at coronary catheterization) to all-cause death or last follow-up. For the CATHGEN dataset, annual, active follow-up for events was conducted by phone and/or mail by trained staff in the Duke Clinical Research Institute. Vital events for the initial dataset were confirmed through the National Death Index, as previously described [45]. For the IMHC dataset, vital events were determined by hospital records and death certificates, and confirmed using Utah Health Department Death certificates and the US Social Security Death Master file, as previously described [44].

### Laboratory Methods

#### SNP selection

The pilot study selected 34 previously-genotyped SNPs that met our quality control metrics for high quality genotyping. For the present study, we evaluated two *LSAMP* SNPs that demonstrated significant hazards of all-cause mortality in our previous pilot study (rs1462845 and rs6788787). [11] These *LSAMP* variants were not in linkage disequilibrium, and were the only two out of seventeen *LSAMP* SNPs with significant hazards in the pilot which were not in LD [11]. SNP characteristics are presented in Table 6.

**Table 6:** *LSAMP* SNP Characteristics.

Gene^^^	SNP	Chromosome	Base-Pair Position^†^	Function	Minor allele	Wild-type allele	MAF
*LSAMP*	rs1462845	3	116,943,010	unknown	G	A	0.34
*LSAMP*	rs6788787	3	116,870,848	unknown	A	G	0.15

SNP, single nucleotide polymorphism; MAF, minor allele frequency.

^**^**^SNPs Located 5’ untranslated region (UTR) of gene (upstream of known *LSAMP* gene).

^**†**^NCBI Build 37.

### DNA Extraction & SNP Genotyping

Genomic DNA was extracted from whole blood using the Puregene system (Gentra Systems, Minneapolis, MN, USA) for CATHGEN samples [13], and the Gentra Autopure LS automated DNA Extractor (Gentra Systems, Minneapolis, MN, USA) for IMHC samples [46]. Genotyping was performed at the Duke Center for Human Genetics genotyping laboratory (Durham, NC, USA) and the Intermountain Heart Institute Genetics laboratory (Salt Lake City, UT, USA). CATHGEN genotyping was performed for 5,566 consecutive CATHGEN participants via allelic discrimination protocol via TaqMan real-time polymerase chain reaction on the 7900 HT platform with Assays from Applied Biosystems (Foster City, CA, USA), details of which have been described elsewhere [47]. For rs1462845, an additional 3,696 individuals were genotyped because this SNP was selected as a quality control marker for laboratory testing due to its excellent genotyping performance. The 384-well plates included a total of 20 quality control samples (eight CEPH [Centre d'Étude du Polymorphisme Humain], eight study sample duplicates, and four no-template controls). SNP mismatches were reviewed by an independent genotyping supervisor for potential genotyping errors as well as checked against previous results, if available. Each SNP had a call frequency across all individuals of at least 95%; each individual had a call rate across all SNPs of at least 95%. IMHC genotyping was similarly performed via Taqman (Applied Biosystems, Foster City, California) on a Vii 7, real-time thermal cycler (Applied Biosystems) for 5,872 consecutive IMHC participants. Five percent of samples were re-genotyped for quality assurance. Successful genotyping was obtained for 94.6% of samples. All evaluated SNPs met our established criteria [48,49] for quality control (QC) Hardy-Weinberg equilibrium (HWE) and linkage disequilibrium (LD).

### Statistical Methods

Statistical analyses were performed using the R program’s Survival package [50]. Initial analyses of the CATHGEN data were followed by replication analyses in the IMHC data. Means and frequencies were calculated for baseline demographic variables, diagnoses and events. Due to the low frequency of non-Caucasians in the replication dataset, we analyzed only self-reported Caucasian participants in all analyses presented. In order to determine CAD-specific genetic effects on survival, we employed case-only Cox multivariate regression models to estimate instantaneous risk (hazard) of all-cause mortality in the context of CAD by genotype groups. These models were censored on number of days from study enrollment (time at coronary catheterization) to all-cause death or last follow-up. Survival probabilities were graphically represented with Kaplan-Meier curves. Due to our preliminary data demonstrating additive genetic effects [11], an additive inheritance model was assumed, assigning wild-type genotypes a value of 0, heterozygous genotypes a value of 1, and risk homozygous genotypes a value of 2 [51]. All SNPs were analyzed separately with a model evaluating only the main effect of genotype (“gene” model) and a separate covariate model controlling for age, sex, body mass index (BMI), and histories of smoking, type 2 diabetes, hyperlipidemia and hypertension (“covariate” model).

We also evaluated robustness to potential confounders by including coronary interventions as a confounder. Coronary intervention was defined as interventional cardiology procedures following initial cardiac catheterization (dichotomous variables indicating presence of subsequent coronary artery bypass graft procedure [CABG] and/or presence of subsequent intra-coronary interventions [ICC] or percutaneous transluminal coronary angiography [PTCA] procedures). Additionally, we conducted stratified analyses by sex, smoking and disease severity (CAD index ≥ 67) as justified by the following: sex/gender differences in cardiovascular survival are well-established; disease severity can impact genetic effects on survival as evidenced by heritability estimates of 45–49% for left-main coronary disease and significantly increased risk of death in unaffected family members [52]; CAD severity explains genetic associations of *LSAMP* with CAD [19]; in pilot analyses of 1,885 CATHGEN participants [11], males with higher burden of CAD demonstrated unique SNP-specific effects on survival probability. Prior results suggested a strong effect of smoking on association with *KALRN* [33]; therefore, we also performed smoking stratified analyses. All models were also evaluated separately in control subjects in order to evaluate whether effects were unique to CAD case status. All Cox models met the proportional hazards assumption, as tested by Grambsch and Themeau’s method [53]

## Supporting Information

S1 Fig **Moving average plots of minor allele frequency for rs6788787 by age deciles in CATHGEN cohort for A) CAD cases and B) controls.** X-axis displays age in deciles. Y-axis displays minor allele frequencies. Middle (black) lines represent calculated frequency. Red lines indicate 95% confidence intervals.
(TIF)

S2 Fig Moving average plots of minor allele frequency for rs6788787 by age deciles in IMHC cohort for A) CAD cases and B) controls. X-axis displays age in deciles. Y-axis displays minor allele frequencies. Middle (black) lines represent calculated frequency. Red lines indicate 95% confidence intervals.
(TIF)

S1 Table Hazards of Death by SNP (Dominant, Recessive) in Caucasian CAD Cases. SNP, single nucleotide polymorphism; MAF, minor allele frequency; CAD, coronary artery disease; HR, hazard ratio; CI, 95% confidence interval.^**^**^Gene model: age, main effect of genotype (additive model). ^**†**^Covariate model: gene, age, body mass index (BMI), histories of hypertension (HTN), type 2 diabetes mellitus (T2DM), hyperlipidemia, smoking.*p < .05, ** *p* < .01
(DOCX)

S2 Table Hazards of Death by SNP (Dominant, Recessive) in Caucasian Males with Severe Burden of CAD. SNP, single nucleotide polymorphism; MAF, minor allele frequency; CAD, coronary artery disease; CADi, CAD index; HR, hazard ratio; CI, 95% confidence interval.^^^Gene model: age, main effect of genotype (additive model).^†^Covariate model: gene, age, body mass index (BMI), histories of hypertension (HTN), type 2 diabetes mellitus (T2DM), hyperlipidemia, smoking.^‡^Intervention model: gene, age, body mass index (BMI), histories of hypertension (HTN), type 2 diabetes mellitus (T2DM), hyperlipidemia, and presence of any of the following subsequent interventional procedures: coronary artery bypass graft surgery (CAGB), percutaneous transluminal coronary angiography (PTCA) or stent. (Note: PTCA or stent not included as covariates in IMHC model due to lack of data.) *p < .05, ** *p* < .01.
(DOCX)

S3 Table Between-genotype tests of association for rs1462845 in Caucasian Males with Severe Burden of CAD. SNP, single nucleotide polymorphism; MAF, minor allele frequency; CAD, coronary artery disease; WT, wild type; HR, hazard ratio; CI, 95% confidence interval.^**^**^Gene model: age, main effect of genotype (additive model). *p < .05, ** *p* < .01
(DOCX)
