# Supplementary figures and images for: Case-Only Survival Analysis Reveals Unique Effects of Genotype, Sex, and Coronary Disease Severity on Survivorship
Source: PLoS One. 2016 May 17;11(5):e0154856. doi: 10.1371/journal.pone.0154856 (PMC4871369; doi:10.1371/journal.pone.0154856)

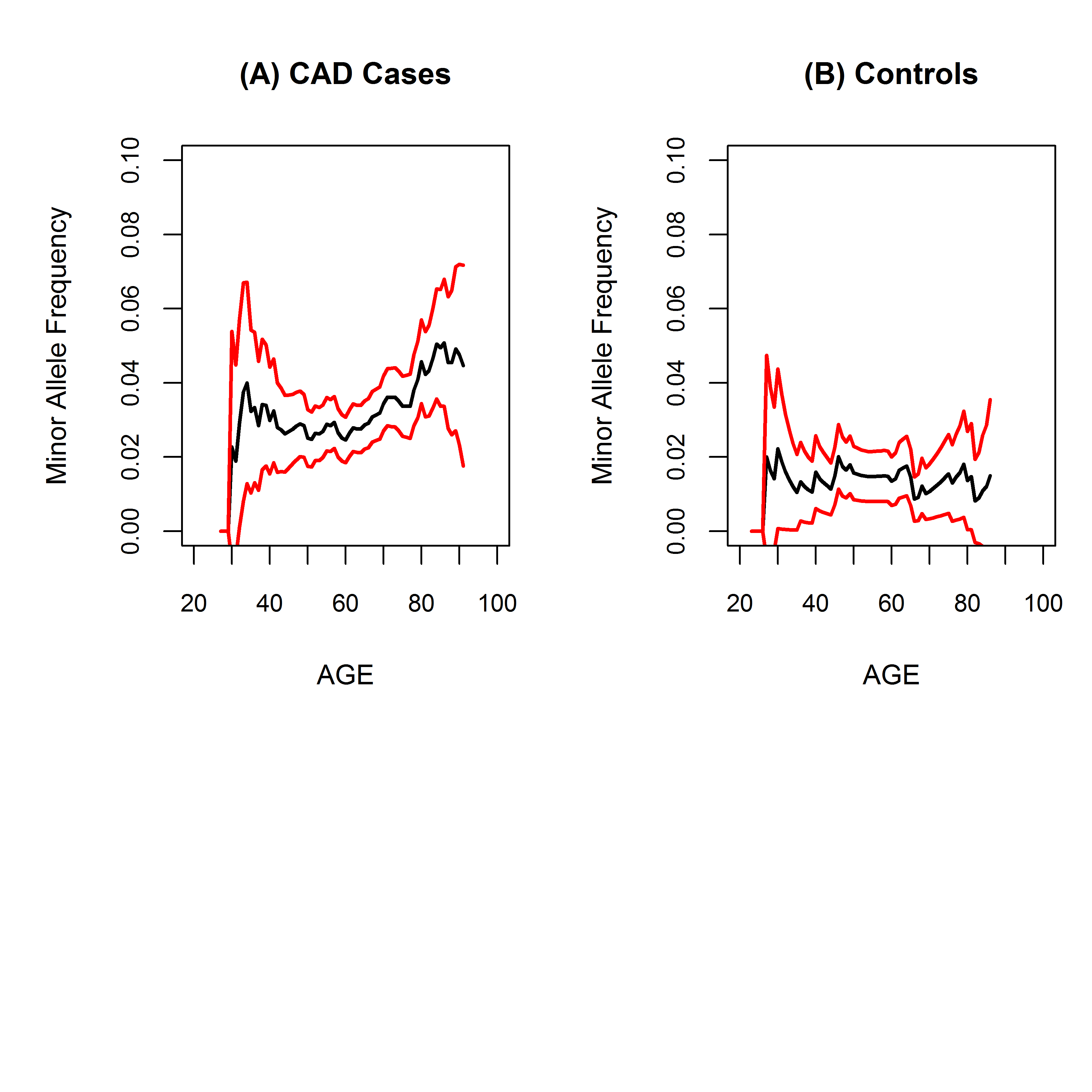

Supplement: S1 Fig — Moving average plots of minor allele frequency for rs6788787 by age deciles in CATHGEN cohort for A) CAD cases and B) controls. X-axis displays age in deciles. Y-axis displays minor allele frequencies. Middle (black) lines represent calculated frequency. Red lines indicate 95% confidence intervals. (TIF) [file pone.0154856.s001.tif]

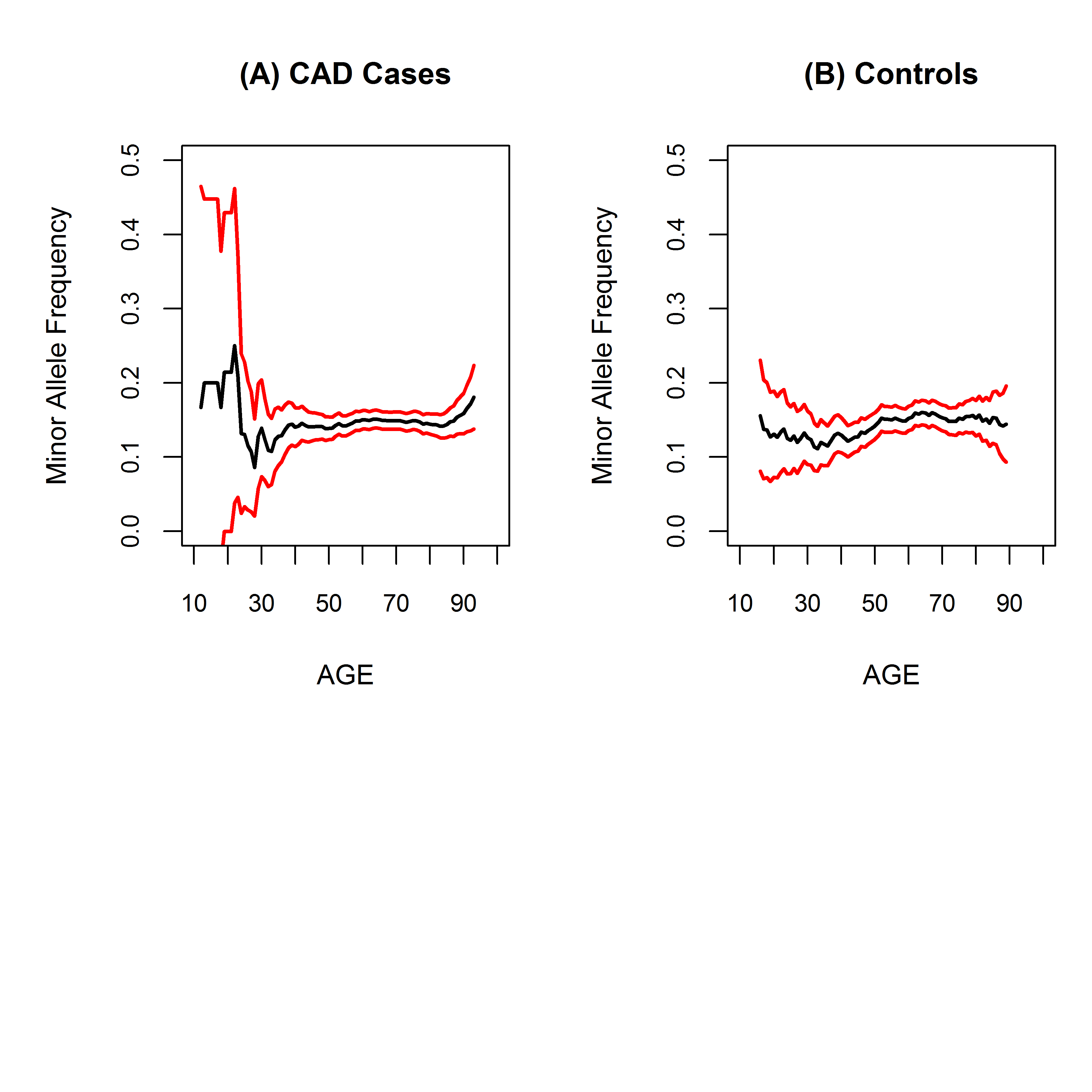

Supplement: S2 Fig — Moving average plots of minor allele frequency for rs6788787 by age deciles in IMHC cohort for A) CAD cases and B) controls. X-axis displays age in deciles. Y-axis displays minor allele frequencies. Middle (black) lines represent calculated frequency. Red lines indicate 95% confidence intervals. (TIF) [file pone.0154856.s002.tif]
