# Supplementary material for: Case-Only Survival Analysis Reveals Unique Effects of Genotype, Sex, and Coronary Disease Severity on Survivorship
Source: PLoS One. 2016 May 17;11(5):e0154856. doi: 10.1371/journal.pone.0154856 (PMC4871369; doi:10.1371/journal.pone.0154856)
Supplement: S1 Table — SNP, single nucleotide polymorphism; MAF, minor allele frequency; CAD, coronary artery disease; HR, hazard ratio; CI, 95% confidence interval.^Gene model: age, main effect of genotype (additive model). †Covariate model: gene, age, body mass index (BMI), histories of hypertension (HTN), type 2 diabetes mellitus (T2DM), hyperlipidemia, smoking.*p < .05, ** p < .01 (DOCX) [file pone.0154856.s003.docx]

**S1 Table. Hazards of Death by SNP (Dominant, Recessive) in Caucasian CAD Cases.**

| **SNP** | **Primary CATHGEN Dataset CAD Cases** | | | | | **Replication IMHC Dataset CAD Cases** | | | | |
| --- | --- | --- | --- | --- | --- | --- | --- | --- | --- | --- |
|  | **N (MAF)** | **Gene model^^^** | | **Covariate model^†^** | | **N (MAF)** | **Gene model^^^** | | **Covariate model^†^** | |
|  |  | **HR (CI)** | ***p*** | **HR (CI)** | ***p*** |  | **HR (CI)** | ***p*** | **HR (CI)** | ***p*** |
| rs1462845 | 6872 (0.338) |  |  |  |  | 4971 (0.350) |  |  |  |  |
| Dominant |  | 1.04 (0.91‒1.17) | 0.592 | 1.08 (0.95‒1.22) | 0.261 |  | 0.93 (0.82‒1.05) | 0.219 | 0.93 (0.82‒1.05) | 0.216 |
| Recessive |  | 1.26 (1.04‒1.51) | **0.016*** | 1.30 (1.08‒1.57) | **0.005**** |  | 1.07 (0.89‒1.28) | 0.493 | 1.09 (0.90‒1.30) | 0.376 |
| rs6788787 | 4063 (0.152) |  |  |  |  | 5043 (0.151) |  |  |  |  |
| Dominant |  | 0.76 (0.64‒0.89) | **0.001**** | 0.75 (0.63‒0.88) | **0.001**** |  | 1.05 (0.92‒1.20) | 0.450 | 1.06 (0.92‒1.21) | 0.422 |
| Recessive |  | 1.03 (0.66‒1.60) | 0.909 | 0.96 (0.62‒1.50) | 0.861 |  | 0.89 (0.60‒1.32) | 0.561 | 0.95 (0.64‒1.42) | 0.817 |

SNP, single nucleotide polymorphism; MAF, minor allele frequency; CAD, coronary artery disease; HR, hazard ratio; CI, 95% confidence interval. **^^^**Gene model: age, main effect of genotype (additive model). **^†^**Covariate model: gene, age, body mass index (BMI), histories of hypertension (HTN), type 2 diabetes mellitus (T2DM), hyperlipidemia, smoking. ****p* < .05, ** *p* < .01**
