# Supplementary material for: Case-Only Survival Analysis Reveals Unique Effects of Genotype, Sex, and Coronary Disease Severity on Survivorship
Source: PLoS One. 2016 May 17;11(5):e0154856. doi: 10.1371/journal.pone.0154856 (PMC4871369; doi:10.1371/journal.pone.0154856)
Supplement: S2 Table — SNP, single nucleotide polymorphism; MAF, minor allele frequency; CAD, coronary artery disease; CADi, CAD index; HR, hazard ratio; CI, 95% confidence interval.^Gene model: age, main effect of genotype (additive model).†Covariate model: gene, age, body mass index (BMI), histories of hypertension (HTN), type 2 diabetes mellitus (T2DM), hyperlipidemia, smoking.‡Intervention model: gene, age, body mass index (BMI), histories of hypertension (HTN), type 2 diabetes mellitus (T2DM), hyperlipidemia, and presence of any of the following subsequent interventional procedures: coronary artery bypass graft surgery (CAGB), percutaneous transluminal coronary angiography (PTCA) or stent. (Note: PTCA or stent not included as covariates in IMHC model due to lack of data.) *p < .05, ** p < .01. (DOCX) [file pone.0154856.s004.docx]

**S2 Table. Hazards of Death by SNP (Dominant, Recessive) in Caucasian Males with Severe Burden of CAD.**

| **SNP** | **Primary CATHGEN Dataset Male CAD Cases, Severe burden (CADi > 67)** | | | | | | | **Replication IMHC Dataset Male CAD Cases, Severe burden (CADi > 67)** | | | | | | |
| --- | --- | --- | --- | --- | --- | --- | --- | --- | --- | --- | --- | --- | --- | --- |
|  | **N (MAF)** | **Gene model^^^** | | **Covariate model^†^** | | **Intervention model^‡^** | | **N (MAF)** | **Gene model^^^** | | **Covariate model^†^** | | **Intervention model^‡^** | |
|  |  | **HR (95 % CI)** | ***p*** | **HR (95 % CI)** | ***p*** | **HR (95 % CI)** | ***p*** |  | **HR (95 % CI)** | ***p*** | **HR (95 % CI)** | ***p*** | **HR (95 % CI)** | ***p*** |
| rs1462845 | 647 (0.35) |  |  |  |  |  |  | 630 (0.35) |  |  |  |  |  |  |
| dominant |  | 1.34 (1.04‒1.71) | **0.0214*** | 1.32 (1.03‒1.69) | **0.0299*** | 1.29 (1.01‒1.66) | **0.0452*** |  | 1.23 (0.97‒1.56) | 0.0814 | 1.29 (1.01‒1.64) | **0.0390*** | 1.24 (0.97‒1.56) | 0.0840 |
| recessive |  | 1.93 (1.40‒2.66) | **0.000065**** | 1.7 (1.22‒2.35) | **0.00159**** | 1.61 (1.15‒2.23) | **0.00495**** |  | 1.45 (1.04‒2.00) | **0.0262*** | 1.63 (1.17‒2.29) | **0.00418**** | 1.54 (1.10‒2.16) | **0.0122*** |
| rs6788787 | 462 (0.15) |  |  |  |  |  |  | 637  (0.14) |  |  |  |  |  |  |
| dominant |  | 0.68 (0.49‒0.93) | **0.0167*** | 0.62 (0.45‒0.86) | **0.00366**** | 0.63 (0.46‒0.87) | 0.00450** |  | 1.01 (0.78‒1.31) | 0.952 | 0.95 (0.73‒1.24) | 0.705 | 1.01 (0.77‒1.31) | 0.9737 |
| recessive |  | 1.41 (0.70‒2.86) | 0.339 | 1.06 (0.52‒2.16) | 0.877 | 1.08 (0.52‒2.21) | 0.841 |  | 1.28 (0.70‒2.34) | 0.420 | 1.09 (0.59‒2.02) | 0.774 | 1.37 (0.74‒2.54) | 0.3158 |

SNP, single nucleotide polymorphism; MAF, minor allele frequency; CAD, coronary artery disease; CADi, CAD index; HR, hazard ratio; CI, 95% confidence interval. ^^^Gene model: age, main effect of genotype (additive model). ^†^Covariate model: gene, age, body mass index (BMI), histories of hypertension (HTN), type 2 diabetes mellitus (T2DM), hyperlipidemia, smoking. ^‡^Intervention model: gene, age, body mass index (BMI), histories of hypertension (HTN), type 2 diabetes mellitus (T2DM), hyperlipidemia, and presence of any of the following subsequent interventional procedures: coronary artery bypass graft surgery (CAGB), percutaneous transluminal coronary angiography (PTCA) or stent. (Note: PTCA or stent not included as covariates in IMHC model due to lack of data.). ****p* < .05, ** *p* < .01.**
