# Supplementary material for: Case-Only Survival Analysis Reveals Unique Effects of Genotype, Sex, and Coronary Disease Severity on Survivorship
Source: PLoS One. 2016 May 17;11(5):e0154856. doi: 10.1371/journal.pone.0154856 (PMC4871369; doi:10.1371/journal.pone.0154856)
Supplement: S3 Table — SNP, single nucleotide polymorphism; MAF, minor allele frequency; CAD, coronary artery disease; WT, wild type; HR, hazard ratio; CI, 95% confidence interval.^Gene model: age, main effect of genotype (additive model). *p < .05, ** p < .01 (DOCX) [file pone.0154856.s005.docx]

**S3 Table. Between-genotype tests of association for rs1462845 in Caucasian Males with Severe Burden of CAD.**

| **SNP** | **Primary CATHGEN Dataset Males, Severe CAD Cases** | | | | | **Replication IMHC Dataset Males, Severe CAD Cases** | | | | |  |
| --- | --- | --- | --- | --- | --- | --- | --- | --- | --- | --- | --- |
|  | **N (MAF)** | **WT vs.Heterozygous^^^** | | **WT vs.Homozygous risk^^^** | | **N (MAF)** | **WT vs.Heterozygous^^^** | | **WT vs.Homozygous risk^^^** | |  |
|  |  | **HR (95% CI)** | ***p*** | **HR (95% CI)** | ***p*** |  | **HR (95% CI)** | ***p*** | **HR (95% CI)** | ***p*** | |
| rs1462845 | 647 (0.35) | 0.87 (0.67–1.12) | 0.27 | 1.56 (1.10–2.22) | 0.013 | 630 (0.35) | 1.18 (0.91–1.54) | 0.22 | 2.11 (1.48–3.01) | **.0000354**** | |

SNP, single nucleotide polymorphism; MAF, minor allele frequency; CAD, coronary artery disease; WT, wild type; HR, hazard ratio; CI, 95% confidence interval. **^^^**Gene model: age, main effect of genotype (additive model). ****p* < .05, ** *p* < .01**
